# Mortality, Hepatic Decompensation, and Cardiovascular- and Renal-Related Outcomes in Lean Versus Non-lean Patients Hospitalized With Metabolic Dysfunction-Associated Steatohepatitis (MASH)

**DOI:** 10.7759/cureus.60968

**Published:** 2024-05-24

**Authors:** Chukwunonso Ezeani, Chidiebele Omaliko, Yazan A Al-Ajlouni, Basile Njei

**Affiliations:** 1 Department of Internal Medicine, Baton Rouge General Medical Center, Baton Rouge, USA; 2 Department of Internal Medicine, Brookdale University Hospital and Medical Center, New York, USA; 3 Department of Rehabilitation, Montefiore Medical Center, Wakefield Campus, New York, USA; 4 Department of Medicine, Yale School of Medicine, New Haven, USA

**Keywords:** metabolic dysfunction-associated steatohepatitis, renal outcomes, lean mash, cardiovascular outcomes, mash

## Abstract

Introduction: Metabolic dysfunction-associated steatohepatitis (MASH) is an important cause of cirrhosis and end-stage liver disease. In addition, there have been reports of worse extrahepatic outcomes, especially cardiovascular events, in patients with lean patients’ fatty liver disease compared to the non-lean group. There is limited data on hepatic, cardiac, and renal outcomes in lean compared to non-lean patients with MASH. This study aims to evaluate the cardiovascular, renal, and hepatic outcomes in hospitalized US adults with MASH, focusing on a comprehensive comparison between lean and non-lean patients.

Methods: The National Inpatient Sample (NIS) database was queried from 2016 to 2020 to identify hospitalizations with MASH. Hospitalizations with a history of overweight and obesity (lean body mass index (BMI) <25 vs. lean BMI >25) were also identified. The primary outcome was in-hospital mortality. Secondary outcomes were major adverse cardiovascular outcomes (MACE: a composite of acute myocardial infarction, cardiac arrest, stroke, heart failure, and atrial fibrillation); major adverse kidney outcome (MAKE: a composite outcome of acute kidney injury (AKI), renal replacement therapy, and renal cancer), and hepatic decompensation (esophageal varices with bleeding, ascites, spontaneous bacterial peritonitis (SBP), hepatic encephalopathy, and hepatorenal syndrome) Multivariate logistic regression analysis was used to derive risk ratios for clinical outcomes.

Results: We included 539,275 MASH patients in our sample; 324,330 (60%) were lean. The included patients were mostly female (61%), the mean age was 64 years, and 76% were White. At baseline, non-lean patients had a higher prevalence of heart failure, hypertension, and hyperlipidemia. There was no difference in the prevalence of smoking among both groups. In a multivariate analysis, with adjustment for age, sex, race, sarcopenia, cardiometabolic risk factors, hospital characteristics, admission type, socioeconomic factors, and all comorbidities (including 31 Elixhauser comorbidities), lean status was associated with a 40% increased risk of mortality (adjusted odds ratio (aOR) 1.40, confidence interval (CI) 1.29-1.53), 19% increased risk of MACE (aOR 1.19; 95% CI 1.14-1.24), 20% increased risk of renal decompensation (aOR 1.25; 95% CI 1.20-1.30), and 33% increased risk of hepatic decompensation (aOR 1.33 CI 1.28-1.38).

Conclusion: Lean patients with MASH are at higher risk of cardiovascular and renal outcomes and may benefit from enhanced screening for early identification and treatment to improve outcomes.

## Introduction

Metabolic dysfunction-associated steatohepatitis (MASH) is increasingly recognized as a leading cause of liver cirrhosis and related complications, with a rising incidence expected to significantly impact public health by 2030 [1]. MASH, characterized by inflammation and cell ballooning, which may progress to fibrosis, poses a particular risk in the context of metabolic syndrome - albeit with a varied prognosis based on patient body mass index (BMI) [2]. MASH with fibrosis has been termed "at-risk" as some patients progress to liver cirrhosis with antecedent hepatic decompensation and hepatocellular carcinoma. In addition, it is important to note that MASH is an increasingly important cause of cirrhosis and is projected to be a leading cause of cirrhosis in the coming years.

Recent studies reveal a paradox where lean patients with MASH, despite generally possessing a favorable metabolic profile, experience disproportionately severe hepatic outcomes and higher mortality rates compared to their non-lean counterparts [3-6]. For instance, although lean patients tend to exhibit less insulin resistance and hepatic fibrosis, they may still progress to advanced liver disease and experience higher rates of liver-related mortality [7]. Moreover, these patients have been linked to an increased risk of both hepatic and extra-hepatic cancers, including pancreatic and colorectal cancers, which compounds the severity of their clinical picture [8].

Despite these severe implications, lean patients with MASH might not present with an elevated risk of developing cardiovascular disease, although their mortality rates from cardiovascular complications remain high [9,10]. This discrepancy points to a complex interplay of genetic, metabolic, and environmental factors that influence disease progression in lean MASH patients [11]. Furthermore, these patients often exhibit a severe histological phenotype that resembles that seen in obese MASH patients, challenging the conventional understanding that links worse liver health outcomes solely to higher body weight [12].

Given the conflicting data and the unique clinical profile of lean MASH patients, this study aims to comprehensively assess how lean status affects clinical outcomes in hospitalized US adults with MASH. Utilizing the National Inpatient Sample (NIS) database, which includes a large, diverse set of hospitalization records across the United States, provides an unprecedented opportunity to examine these outcomes on a national scale. To the best of our knowledge, this is the first study to employ the NIS database to investigate the influence of lean versus non-lean status on the progression and outcomes of MASH in a hospitalized population. By focusing on patients with active inflammation, with or without fibrosis, our research seeks to clarify the implications of BMI on the progression and outcomes of MASH. This approach not only enhances our understanding of how BMI interacts with metabolic liver disease but also contributes critical insights into its management and prognosis, thus addressing a significant gap in the existing literature.

## Materials and methods

Study design

This study conducted a retrospective analysis of prospectively collected data from the NIS, the largest hospital-related dataset in the United States. The NIS documents in-hospital events, including diagnosis and outcomes.

Data collection

We queried the NIS database for the years 2016 to 2020. Data in the NIS are coded using the International Classification of Diseases (ICD), version 10, and include patient demographic details, such as age, BMI, and gender. In-hospital outcomes including mortality are also recorded.

Study variables

Patients admitted with a diagnosis of MASH were identified. These patients were stratified based on the BMI into lean (BMI < 25 kg/m^2^) and non-lean (BMI ≥ 25 kg/m^2^) groups. Comorbidities, such as diabetes mellitus, hypertension, hyperlipidemia, cardiac arrhythmia, alcohol abuse, valvular heart disease, pulmonary circulation disorders, peripheral vascular disease, and sarcopenia, were identified using ICD-10 codes.

Study outcomes

Primary outcomes included hepatic decompensation events, such as variceal bleeding, ascites, spontaneous bacterial peritonitis, hepatic encephalopathy, and hepatorenal syndrome. Major adverse cardiovascular events (MACEs), like cardiac arrest, heart failure, myocardial infarction, cerebrovascular accident, and atrial fibrillation, were also assessed. Renal decompensation outcomes, including acute kidney injury, renal replacement therapy, and kidney cancer, were extracted. Composite outcomes of hepatic decompensation, MACE, and renal decompensation were evaluated.

Statistical analysis

Analysis was performed using Stata Statistical Software: release 17 (StataCorp., 2021, College Station, TX: StataCorp LLC). The total number of patients with MASH was generated and stratified based on the BMI into lean versus non-lean categories. A univariate analysis was conducted initially, and variables with a P-value <0.2 were included in the multivariable analysis. The impact of lean status on hepatic, MACE, and renal decompensation was analyzed using multivariate regression for continuous variables and logistic regression for binary or categorical variables.

Ethical consideration

As the NIS contains de-identified data, this study was exempt from Institutional Review Board (IRB) review.

## Results

During the study period spanning from 2016 to 2020, we identified a total of 539,275 hospital admissions featuring a diagnosis of metabolic MASH. Lean patients accounted for 60% (n = 324,330) of this population, while the remaining 40% (n = 214,945) were classified as non-lean. The demographic breakdown revealed a mean age of 64 years in the lean group versus 58.9 years in the non-lean group, with a predominance of female patients in both cohorts. The racial distribution was largely white, with blacks representing the least common ethnic group among the study population. A high frequency of comorbid metabolic conditions, such as diabetes, hypertension, and hyperlipidemia, were observed, with most patients being covered by private insurance and treated in urban teaching centers (Table 1).

**Table 1:** Demographic and clinical characteristics of hospitalized patients with MASH, stratified by the BMI category MASH: metabolic dysfunction-associated steatohepatitis, BMI: body mass index

Variable	Lean (n = 324,330; 60%)	Non-lean (n = 214,945; 40%)	P-value
Age, mean (years)	64.0	58.9	
Gender			<0.001
Female	60.6%	63.6%	
Male	39.4%	36.4%	
Race/ethnicity			<0.001
White	76.6%	76.8%	
Black	4.1%	5.1%	
Hispanic	13.7%	13.4%	
Other	5.7%	4.6%	
Charlson comorbidity index ≥3	74.2%	67.3%	<0.001
Comorbidities			
Diabetes mellitus	62.6%	64.1%	<0.001
Hypertension	67.9%	71.8%	<0.001
Hyperlipidemia	38.7%	42.7%	<0.001
Smoking	0.6%	0.5%	0.0347
Sarcopenia			
Type of admission			<0.001
Elective admission	9.7%	16.3%	
Weekend admission	22.1%	20.7%	
Primary payer source			<0.001
Private insurance	61.1%	50.3%	
Medicaid	11%	14.1%	
Medicare	23%	30.3%	
Other payment source	2.5%	3.0%	
Self-pay	0.2%	0.2%	
No charge	2.2%	2.2%	
Median household income, $			<0.001
<45,999	29.8%	30.0%	
46,000-58,999	28.2%	29.1%	
59,000-78,999	24.5%	25.4%	
>79,000	17.4%	15.6%	
Hospital location status			<0.001
Rural	8.1%	6.9%	
Urban	91.9%	93.1%	
Hospital teaching status			0.041
Teaching	73.9%	74.9%	
Non-teaching	26.1%	25.1%	
Hospital bed size			<0.001
Small	17.0%	18.4%	
Medium	25.4%	25.5%	
Large	57.6%	56.1%	

Liver-related outcomes

**Figure 1:**
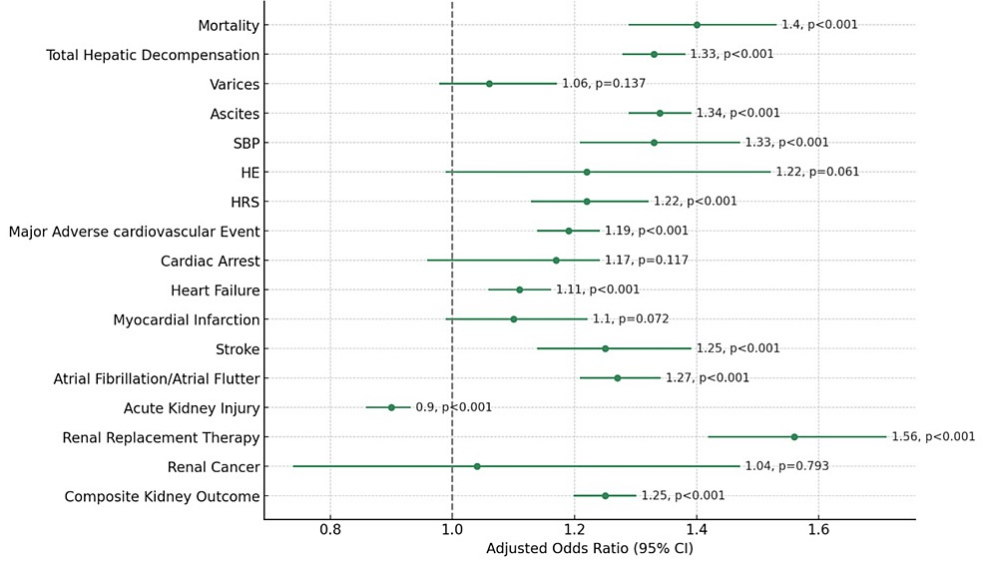
Risk of mortality and decompensation outcomes in lean versus non-lean patients with MASH This forest plot illustrates the adjusted odds ratios (ORs) with 95% confidence intervals (CIs) for mortality, hepatic, cardiovascular, and renal decompensation outcomes in a hospitalized cohort of patients with metabolic dysfunction-associated steatohepatitis (MASH), stratified by lean and non-lean status. The plot highlights the OR for each outcome, comparing lean versus non-lean individuals, adjusted for potential confounders. Points to the right of the vertical line at OR = 1 suggest an increased risk in lean patients, while points to the left suggest a decreased risk. The accompanying p-values, displayed in brackets next to the ORs, indicate the statistical significance of each finding, supporting the conclusion that lean patients with MASH exhibit a distinct profile of health risks.

Ascites emerged as the most prevalent hepatic decompensation event, observed in 23-30.9% of cases. Lean patients with MASH had a significantly increased adjusted odds ratio (aOR) for developing ascites at 1.34 (95% CI 1.29-1.39; p < 0.001), as well as for spontaneous bacterial peritonitis (aOR 1.33; CI 1.21-1.47; p < 0.001), and hepatorenal syndrome (aOR 1.22; CI 1.13-1.32; p < 0.001). While the risk for variceal bleeding and hepatic encephalopathy did not differ significantly between the lean and non-lean groups, the composite measure of hepatic decompensation was notably higher in the lean group (aOR 1.33; 95% CI 1.28-1.38; p < 0.001), indicating a trend toward more severe liver-related events in this population (Table 2, Figure 1).

**Table 2:** Adjusted odds ratios for cardiovascular, renal, mortality, and decompensation outcomes comparing lean versus non-lean patients with MASH ^a^Adjusted for for age, sex, race, sarcopenia, cardiometabolic risk factors, hospital characteristics, admission type, socioeconomic factors, and all comorbidities (including 31 Elixhauser comorbidities). Abbreviations: SBP: spontaneous bacterial peritonitis; HE: hepatic encephalopathy; HRS: hepatorenal syndrome; OR: odds ratio

Outcomes	Lean (%)	Non-lean (%)	Unadjusted OR (95% CI)	Adjusted OR (95% CI)^a^	P-value
Mortality, No (%)	4.1%	3.2%	1.28 (1.20-1.37)	1.40 (1.29-1.53)	<0.001
Liver-related outcomes					
Total hepatic decompensation (%)	35.7%	27.3%	1.48 (1.43-1.53)	1.33 (1.28-1.38)	<0.001
Varices	4.2%	2.9%	1.46 (1.36-1.56)	1.06 (0.98-1.17)	0.137
Ascites	30.9%	23.3%	1.47 (1.43-1.52)	1.34 (1.29-1.39)	<0.001
SBP	3.4%	2.3%	1.51 (1.40-1.64)	1.33 (1.21-1.47)	<0.001
HE	0.53%	0.45%	1.16 (0.97-1.39)	1.22 (0.99-1.52)	0.061
HRS	5.0%	4.2%	1.19 (1.11-1.27)	1.22 (1.13-1.32)	<0.001
Cardiac-related outcomes					
Major adverse cardiovascular event	30.4%	33.0%	0.91 (0.89-0.94)	1.19 (1.14-1.24)	<0.001
Cardiac arrest	0.64%	0.64%	1.01 (0.86-1.18)	1.17 (0.96-1.24)	0.117
Heart failure	22.5%	26.4%	0.81 (0.79-0.84)	1.11 (1.06-1.16)	<0.001
Myocardial infarction	2.6%	2.3%	1.15 (1.06-1.25)	1.10 (0.99-1.22)	0.072
Stroke	0.11%	0.01%	1.40 (1.29-1.51)	1.25 (1.14-1.39)	<0.001
Atrial fibrillation/atrial flutter	13.0%	12.5%	1.05 (1.01-1.08)	1.27 (1.21-1.34)	<0.001
Kidney-related outcomes					
Acute kidney injury	31.4%	30.1%	1.06 (1.03-1.10)	0.90 (0.86-0.93)	<0.001
Renal replacement therapy	4.3%	3.6%	1.21 (1.13-1.30)	1.56 (1.42-1.71)	<0.001
Renal cancer	3.2%	2.8%	1.13 (1.06-1.22)	1.04 (0.74-1.47)	0.793
Composite kidney outcome	34.2%	31.9%	1.11 (1.07-1.41)	1.25 (1.20-1.30)	<0.001

Cardiac-related outcomes

In terms of cardiovascular events, heart failure was the most frequently documented major adverse cardiovascular event (MACE) in our cohort. Lean patients were at an increased risk for heart failure (aOR 1.11; 95% CI 1.06-1.16; p < 0.001), stroke (aOR 1.25; 95% CI 1.21-1.34; p < 0.001), and atrial fibrillation/flutter (aOR 1.27; 95% CI 1.21-1.34; p < 0.001). The incidence of cardiac arrest and myocardial infarction was statistically similar across both groups. The analysis also indicated that lean patients had an approximately 20% greater risk of experiencing composite MACE outcomes compared to non-lean patients (aOR 1.19; 95% CI 1.14-1.24; p < 0.001) (Table 2, Figure 1).

Kidney-related outcomes

The most common renal adverse outcome was the development of AKI, with lean patients being slightly less susceptible to AKI during hospitalization (aOR 0.90; 95% CI 0.86-0.93; p < 0.001). However, the likelihood of needing renal replacement therapy was substantially higher in the lean group (aOR 1.56; 95% CI 1.42-1.71; p < 0.001). No significant difference was observed in the rates of renal cell cancer between lean and non-lean patients. Importantly, when considering the composite risk of adverse kidney-related outcomes, lean patients were found to be at a higher risk compared to their non-lean counterparts (aOR 1.25; 95% CI 1.20-1.30; p < 0.001) (Table 2, Figure 1).

## Discussion

This nationwide study aimed to investigate the differences in the risk profiles of hepatic, cardiovascular, and renal outcomes between lean and non-lean adults with MASH across a substantial US population sample. Analyzing data from 539,275 hospitalizations, our research reveals significant disparities. Lean individuals were notably more likely to develop severe hepatic complications, such as ascites, spontaneous bacterial peritonitis, and hepatorenal syndrome. In addition, these patients faced heightened risks for serious cardiovascular and renal events, including heart failure, stroke, atrial fibrillation, and the necessity for renal replacement therapy during their hospital stay. These findings challenge the traditional view that associates higher body mass with increased severity of metabolic diseases and underscore the severe disease trajectory in lean individuals with MASH. By demonstrating that lean patients may experience a more adverse disease course, our study addresses a critical gap in the medical literature and prompts further investigation into the unique pathophysiological mechanisms driving these outcomes in lean populations.

Our findings corroborate and extend the results of previous studies on patients with lean metabolic dysfunction-associated steatohepatitis (MASLD), underscoring the heightened risk profile in this subgroup. Population-based studies, including those by Nabi et al. and Almomani et al., have demonstrated increased rates of fibrosis, liver-related adverse events, and cardiovascular complications, such as acute coronary syndrome and chronic kidney disease, highlighting a serious health burden even in lean populations [13,14]. Moreover, a recent systematic review by Ha et al., encompassing 10 studies, revealed an elevated risk of liver-related mortality in the lean MASLD group, although risks for all-cause mortality and cardiovascular mortality were comparable to those in non-lean groups [15]. In addition, a comprehensive meta-analysis by Huang et al., across 12 studies, indicated an increased risk of all-cause mortality in lean MASLD patients [16]. Complementing these studies, recent research has evaluated cardiovascular outcomes in lean versus non-lean MASLD cohorts, calculating pooled ORs for various outcomes. This meta-analysis found a 50% increase in the odds of cardiovascular mortality for lean MASLD [17]. These findings emphasize the critical need for a nuanced understanding of MASLD in lean individuals, challenging the traditional paradigms that predominantly associate severe liver disease outcomes with obesity. Our study contributes novel insights by quantifying these risks in a large cohort, reinforcing the imperative for targeted screening and intervention strategies that are specifically tailored to the unique risk profiles of lean individuals with MASLD.

Our findings indicate that lean status in patients with MASH is associated with significantly increased risks of mortality, MACE, renal decompensation, and hepatic decompensation. Specifically, lean MASH patients exhibited a 40% increased risk of mortality, a 19% increased risk of MACE, a 20% increased risk of renal decompensation, and a 33% increased risk of hepatic decompensation. These elevated risks may be attributable to a combination of visceral adiposity, metabolic dysfunction despite a normal BMI, and a "metabolically obese, normal weight" phenotype, which predisposes these patients to insulin resistance, dyslipidemia, and systemic inflammation. Furthermore, unique pathophysiological mechanisms, such as more pronounced hepatic insulin resistance and endothelial dysfunction, may exacerbate disease severity in these individuals [18,19]. Delays in diagnosis and treatment, often due to misconceptions about the risk associated with body weight, may allow the disease to progress to more severe stages [20,21]. Genetic predispositions affecting fat distribution and lipid metabolism, along with distinct inflammatory and immune profiles, may also contribute to the heightened severity of clinical outcomes observed in lean individuals [18,22]. These findings challenge existing paradigms and highlight the need for tailored diagnostic and therapeutic approaches that consider the specific risk profiles and pathophysiological bases of disease in lean MASH patients, urging further research into their unique genetic and metabolic landscapes.

Clinical, public health, and policy implications

Our findings necessitate a reevaluation of current clinical practices concerning MASH. Traditionally, screening protocols have focused on individuals with higher BMIs; however, our study highlights the critical need for including lean individuals in these assessments. Clinicians must be aware that lean patients with MASH may experience severe disease courses, necessitating tailored treatment strategies and vigilant monitoring for cardiovascular, renal, and hepatic complications.

In addition to clinical considerations, public health initiatives should prioritize educating the public on the risks of MASH among lean individuals. Awareness campaigns are essential to dispel the common misconception that metabolic diseases are exclusive to overweight or obese populations. In addition, integrating liver health screenings within community health programs, particularly those addressing cardiovascular health, could facilitate early detection and intervention, thereby improving outcomes for at-risk populations.

Furthermore, the findings of this research support the development of health policies that ensure metabolic disease screenings are inclusive of all body types. Health insurance coverage should be expanded to include comprehensive metabolic screenings for individuals regardless of BMI, thus reducing the economic barriers to accessing such healthcare services. Furthermore, policymakers should consider increasing funding for research focused on understanding the pathophysiological differences in MASH across different body compositions to inform more effective treatments.

Finally, to enhance the effectiveness of medical training, educational curricula should include detailed information on the diagnosis and management of MASH in lean individuals. Continuing medical education (CME) programs should be updated to include the latest research findings and treatment approaches for MASH, ensuring that healthcare providers are well-equipped to manage this complex disease across all patient demographics.

Strengths and limitations

Our study makes several notable contributions to the understanding of MASH in lean individuals. First, it is pioneering in highlighting the increased risk of cardiac, renal, and hepatic decompensation within this specific subgroup, marking a significant step forward in identifying high-risk populations. This insight is critical for developing targeted screening strategies and tailored intervention protocols. In addition, the use of the NIS, the largest publicly available all-payer inpatient care database in the US, allowed for a robust analysis due to its size and diversity, enhancing the reliability of our findings. This comprehensive dataset enabled us to assess a broad spectrum of complications associated with MASH, thereby underscoring the multifaceted impact of the disease in lean patients.

Despite these strengths, our study has several limitations that should be acknowledged. The reliance on ICD-10 coding within the NIS database introduces potential biases due to miscoding or misclassification of patient data. While this system allows for widespread data collection and analysis, the accuracy of diagnostic coding can vary, potentially affecting the precision of our outcome assessments. Furthermore, as our analysis was confined to the US population, the findings may not be directly applicable to other global regions with different demographic and health profiles. Therefore, conducting similar studies in diverse international settings would be crucial to validate and potentially generalize our results. In addition, the retrospective nature of the data limits our ability to ascertain causal relationships, and prospective studies would be beneficial to further elucidate the mechanisms underlying the observed associations.

Future research

The significant associations between lean status and increased risks of cardiac, renal, and hepatic decompensation highlighted in our study raise important questions for future research. Particularly, it becomes crucial to explore the pathophysiological mechanisms that may predispose lean individuals with MASH to more severe outcomes. Longitudinal studies investigating the metabolic profiles, inflammatory markers, and genetic predispositions of this demographic could provide deeper insights into their unique disease progression patterns. In addition, interventional studies designed to test the efficacy of specific therapeutic approaches tailored for lean MASH patients could inform more personalized management strategies. Investigating the impact of lifestyle modifications, pharmacotherapy, and surveillance intervals in altering the disease trajectory in lean versus non-lean populations will also be vital. Such research could ultimately lead to more refined and effective clinical protocols that better address the nuances of MASH in lean individuals.

## Conclusions

This study conclusively demonstrates, utilizing the NIS dataset, that hospitalizations involving lean patients with MASH are significantly associated with heightened risks of cardiac, renal, and hepatic decompensation compared to their non-lean counterparts. These findings emphasize the critical need for vigilant cardiovascular and renal monitoring in this patient demographic, suggesting that lean MASH patients may exhibit a more severe disease course requiring proactive management strategies. Moreover, our results advocate for a reevaluation of current clinical guidelines to incorporate tailored screening and therapeutic approaches that address the unique risks faced by lean individuals with MASH. This could potentially improve patient outcomes and mitigate the higher morbidity and mortality risks identified in our analysis. Future research should focus on longitudinal studies to unravel the underlying mechanisms driving the increased risk in lean MASH patients, paving the way for more effective targeted interventions.
